# Antivirulent Properties of Underexplored *Cinnamomum tamala* Essential Oil and Its Synergistic Effects with DNase against *Pseudomonas aeruginosa* Biofilms – An *In Vitro* Study

**DOI:** 10.3389/fmicb.2017.01144

**Published:** 2017-06-26

**Authors:** Sanaulla Farisa Banu, Durairajan Rubini, Sairam Rakshitaa, Kamaraj Chandrasekar, Ramar Murugan, Aruni Wilson, Shanmugaraj Gowrishankar, Shunmugiah Karutha Pandian, Paramasivam Nithyanand

**Affiliations:** ^1^Biofilm Biology Laboratory, School of Chemical and Biotechnology, SASTRA UniversityThanjavur, India; ^2^Govind Ballabh Pant National Institute of Himalayan Environment and Sustainable DevelopmentAlmora, India; ^3^School of Chemical and Biotechnology, SASTRA UniversityThanjavur, India; ^4^Division of Microbiology and Molecular Genetics, School of Medicine, Loma Linda University, Loma LindaCA, United States; ^5^Department of Biotechnology, Science CampusKaraikudi, India; ^6^Centre for Research on Infectious Diseases, School of Chemical and Biotechnology, SASTRA UniversityThanjavur, India

**Keywords:** biofilm, *Cinnamomum* essential oil, synergism, DNase, Almora

## Abstract

*Pseudomonas aeruginosa* is a nosocomial pathogen colonizing patients with chronic infectious diseases and has gained resistance to all the known broad spectrum antibiotics available today. The present study showcases the antibiofilm potential of an essential oil (EO) from an underexplored *Cinnamomum* species namely, *C. tamala*, against *P. aeruginosa* biofilms. Furthermore, the synergistic effects of the EO along with a commercially available DNase (DNaseI) and a DNase (MBD) isolated from a marine bacterium were explored for its antibiofilm activity. The results showed that the synergized action has maximum efficacy in inhibiting young and preformed biofilms. The synergized effect of EO and DNaseI showed 70% inhibition against matured biofilms of *P. aeruginosa*. The EO from *C. tamala* also showed quorum sensing inhibitory potential as it could inhibit the swarming motility behavior of *P. aeruginosa*. The synergistic action of EO and DNases offers a novel alternate therapeutic strategy for combating *P. aeruginosa* biofilm associated infections.

## Introduction

Essential oils (EOs) are natural plant secondary metabolites which constitute a complex mixture of volatile components found to have multiple antimicrobial properties (Ogunwande et al., 2005; Matan et al., 2006; Millezi et al., 2013). EOs containing aldehydes or phenols, such as cinnamaldehyde, citral, carvacrol, eugenol, and thymol as major components have exhibited the highest antibacterial activity, wherein the phenolic components present in the EOs are considered to be responsible for the antimicrobial property (Bassole and Juliani, 2012). Since the action of EOs and their constituents bring forth research into the development of novel biocides with broad spectrum activity to combat antimicrobial resistance, several studies are taking place worldwide in bioprospecting EOs for its anti-virulent properties (Gupta et al., 2013; Nazzaro et al., 2013; Utchariyakiat et al., 2016).

*Pseudomonas aeruginosa* is a notorious nosocomial pathogen which is ubiquitous in health care settings where it causes persistent and chronic infection in immunocompromised individuals (Sydnor and Perl, 2011). A majority of *P. aeruginosa* infections are difficult to treat since the bacterial cells protect themselves by a self-produced exopolymeric matrix called biofilms (Ribeiro et al., 2016). The exopolysaccharide (EPS) matrix of both bacterial and fungal biofilms delays the infiltration of antimicrobial agents and act as a barrier thus endorsing intrinsic resistance to several antibiotics which leads to recalcitrant infections (Chung and Toh, 2014; Nithyanand et al., 2015a,b; Frieri et al., 2016). Proteins, nucleic acids and lipids make up the major portion of the biofilm matrix and recently the presence of extracellular DNA (eDNA) has also been documented within the biofilm matrix (Okshevsky and Meyer, 2015). eDNA helps in the adhesion of the biofilm on to any solid surface (Regina et al., 2014), promotes bacterial aggregation (Das and Manefield, 2012) and stabilizes the biofilm architecture (Kreth et al., 2009). Most importantly, a recent study shows that eDNA solely supports biofilm formation by *P. aeruginosa* which caused Catheter associated Urinary Tract Infection (Cole et al., 2014). Biofilm formation and swarming motility are quorum sensing (QS) controlled virulence factors in *P. aeruginosa*. The release of eDNA and swarming motility is mediated through QS dependent mechanism involving *N*-acyl homoserine lactones (AHL) and *Pseudomonas* quinolone signaling (PQS) molecule (Rasamiravaka et al., 2015). Swarming motility is responsible for surface colonization and dissemination of the pathogen which contribute to the formation of biofilms (O’May and Tufenkji, 2011). The failure of antimicrobial therapy to eliminate biofilm associated infections (Davies, 2003) has pushed the scientific fraternity to look for alternative solutions to suppress this key virulent trait of pathogens. Natural compounds from plants and other sources have currently gained importance (Koh et al., 2013) and EOs from aromatic plants is considered as alternative antimicrobial and antibiofilm agents (Nithyanand et al., 2015c). Since deoxyribonucleases have been used to degrade eDNA in biofilms (Pressler, 2008; Nijland et al., 2010), we envisioned that the combination of EO and DNases might have an enhanced biofilm disruption activity. So the present study focuses on the exploration of the synergistic effects of the EO of *Cinnamomum tamala* (Buch.-Ham.) T. Nees and Eberm (Indian bay leaf/Indian cassia) and DNases to disrupt biofilms. The literature scrutiny reveals that till date, the antibiofilm potential of only the commercial cinnamon/Ceylon cinnamon (*Cinnamomum zeylanicum*) has so far been reported (Gupta et al., 2013; Kalia et al., 2015). The present study is the first of its kind to showcase the biofilm inhibition as well as QS inhibition potential of *C. tamala*, which broadens the pharmacological prospects of this wild species and the synergistic effects of EO with DNases have better biofilm disruption ability.

## Materials and Methods

### Test Organism

*Pseudomonas aeruginosa* (PAO1) wild-type strain and two clinical *P. aeruginosa* isolates AU07 (GenBank Accession No: JN871909) and AU09 (GenBank Accession No: JN871910) (Sethupathy et al., 2016) which were obtained as a generous gift from Dr. S. Karutha Pandian, Alagappa University were maintained in Luria Bertani (LB) agar (HiMedia, India). Prior to the experiment, the cultures were grown overnight in LB broth in Orbital shaker at 150 rpm, at 37°C. From this, 100 μl of the suspension containing 10^7^ cells ml^-1^ was used for all the assays (Musthafa et al., 2011).

### Source of Enzymes

Bovine pancreatic DNaseI was purchased from HiMedia and Marine Bacterial DNase (MBD) was isolated and purified from the marine bacterium *Vibrio alginolyticus* (GenBank Accession Number: KX458518).

#### Source and Analyses of Essential Oil

The leaves of *C. tamala* were collected from Almora, Uttarakhand, India. The leaves were partially shade dried and used for extraction of EO. On hydro-distillation, the partially shade dried leaves yielded 1.2% pale yellow color EO. After distillation, the EO was dried over by adding a pinch of anhydrous sodium sulfate and stored at 4°C until further analysis and experiments. The EO was quantitative and qualitatively analyzed by GC-FID and GC-MS following the methods and conditions used by Sriramavaratharajan et al. (2016). The individual component of the EO such as cinnamaldehyde (#W228613) and linalool (#L2602) was purchased from Sigma–Aldrich.

#### Determination of Minimum Inhibitory Concentration (MIC)

The Minimum Inhibitory Concentration (MIC) of the EOs were determined as per Clinical and Laboratory Standards Institute (2006) guidelines. Various concentrations of EO were prepared by mixing proportions of EO with 1.25% dimethyl sulfoxide (DMSO). Serial two-fold dilutions of the EO ranging from 1 to 20% v/v were prepared in 96-well titer plate supplemented with test culture and incubated for 24 h at 37°C. Following incubation, microtiter plates were read spectrophotometrically at 620 nm. The lowest concentration inhibiting the growth of the pathogen was determined as the MIC. Similarly, the individual component of EO such as cinnamaldehyde and linalool was tested for their respective MICs against PAO1 and the clinical isolates.

#### Growth Curve Analysis

In order to examine that the EO did not have bactericidal effect against *P. aeruginosa* planktonic cells, growth curve analysis was done. LB broth containing 1% overnight culture of *P. aeruginosa* was supplemented with Biofilm Inhibitory Concentration (BIC) of the EO and the flask was incubated for 24 h at 37°C. The flask containing overnight culture without EO served as control. The optical density was read spectrophotometrically at every 1 h interval up to 24 h (Packiavathy et al., 2013).

### Antibiofilm Activity of EO, DNases

#### Biofilm Inhibition Assay

The effect of EO to inhibit the young biofilms was assessed using the crystal violet assay (Nithyanand et al., 2015c). The assay was carried out in 24-well microtiter plate (NEST Biotechnology, Korea) containing 1ml of media, 100 μl of cell suspension (10^7^ cells ml^-1^) and EO at its sub-MIC (5% v/v). The well without EO and only DMSO (1.25%) served as a control. Likely, the effect of commercial and MBDs and synergism of EO and the respective DNases were assessed as above to determine the maximum efficacy in inhibiting the biofilm.

$$\text{Percentage Inhibition = }[\frac{\text{Control OD at 595 nm - Test OD at 595 nm}}{\text{Control OD at 595 nm}}]\times 100$$

#### Matured Biofilm Disruption Ability of EO and DNases

To determine the potency of the EO to inhibit mature biofilms, the BIC of EO was added to the preformed biofilms and incubated further for 24 h at 37°C. After incubation, the spent media was discarded and the wells were washed with sterile distilled water. After air drying the wells were stained with 0.4% crystal violet and the absorbance was quantified spectrophotometrically at 595 nm (Nithyanand et al., 2015c). Similarly, the potency of DNases and synergism of EO and DNases over mature biofilm dispersal was also determined.

### Microscopic Visualization of Antibiofilm Activity

#### Light Microscopy

Biofilms were allowed to form on 1 cm × 1 cm glass slides which were placed into the wells of the 24-well titer plates. The BIC of EO (5%v/v) was added to the wells and the plates were incubated at 37°C for 24 h. After incubation, the biofilm formed on the glass slides was stained using crystal violet dye for 5 min. It was then gently washed with de-ionized water and allowed to dry for 5 min. Then, the slides were viewed under light microscope at a magnification of 40× (Nikon Eclipse Ti 100, Japan) (Thenmozhi et al., 2009).

#### Confocal Laser Scanning Microscopy (CLSM)

Confocal Laser scanning microscopy (CLSM) was used to determine the action of EO on mature biofilms. The biofilms were allowed to form on 1 cm × 1 cm glass slide placed in a 24-well titer plates. After 24 h incubation, the BIC of EO was added to the preformed biofilms and the plates were further incubated at 37°C for 24 h. The three dimensional architecture of the preformed biofilm treated with BIC of EO was assessed by CLSM (Nithyanand et al., 2015c). Images were captured and processed by using Zeiss LSM Image Examiner Version 4.2.0.121. The parameters such as biomass, average thickness and surface volume ratio of the control and treated biofilms were evaluated using COMSTAT software (Heydorn et al., 2000).

#### Scanning Electron Microscopy (SEM)

In order to visualize the reduction in EPS of *P. aeruginosa* biofilm, SEM analysis was performed. Briefly, *P. aeruginosa* biofilms were grown in the presence and absence of enzymes (DNaseI and MBD) on the glass slides. The biofilms on the glass slides were fixed with 2.5% glutaraldehyde were washed in 0.1M sodium acetate buffer (pH 7.3). Finally, the dehydrated samples were dried, gold sputtered and examined with VEGA3 TESCAN (Thenmozhi et al., 2009).

#### eDNA Staining

In order to investigate the accumulation of eDNA in *P. aeruginosa* biofilm, eDNA staining was performed. The *P. aeruginosa* strains were inoculated in LB broth and grown in the presence of DNaseI and MBD onto 1 cm × 1 cm glass slide within a 24-well titer plate and was incubated for 24 h at 37°C. After 24 h, the glass slides were gently rinsed with PBS and stained with 20 μM propidium iodide (PI) (Sigma), which stains the DNA present within a biofilm. Accumulation of eDNA were visualized under a fluorescence microscope (Nikon eclipse Ni, Japan) at excitation and emission wavelengths of 540 and 525 nm for PI and photographed digitally (Rajendran et al., 2013).

### Virulence Assays

All the QS mediated virulence assays were performed with the EO against *P. aeruginosa* (PA01) as well as the clinical *P. aeruginosa* isolates. In addition, cinnamaldehyde and linalool which were the major components of EO were also assayed individually against the QS mediated virulence factors of PA01 and the clinical isolates.

#### EPS Inhibition Assay

Bacterial biofilm consists of self-generated extra polymeric substances which plays a distinct role during biofilm formation. EPS from EO treated and untreated test pathogen was quantified by the method of Nithya et al. (2011). The test pathogen was grown in glass slides placed in 24 well microtiter plates with and without the addition of EO and incubated for 16 h. The glass slides were removed and washed with 0.5 ml of 0.9% NaCl. Equal volume of 5% phenol was added to the cell suspension, to which five volume of concentrated H_2_SO_4_ was also added. The mixture was incubated for 1 h in the dark and centrifuged at 10,000 rpm for 10 min. The absorbance of the supernatant was measured at 490 nm.

#### Alginate Assay

Alginate is an abundant polysaccharide in *P. aeruginosa* biofilms which aids in the adhesion of EPS that promote cell attachment and shields the cell surface structure. Alginate was quantified from the EO treated and untreated cell suspension. 600 μl of boric acid – H_2_SO_4_ (4: 1) was added to 70 μl cell suspension and the cell suspension was vortexed for 10 s. Further, 20 μl of 0.2% carbazole solution was added to the cell suspension which was incubated for 30 min at 55°C. After incubation, absorbance was measured spectrophotometrically at 530 nm (Owlia et al., 2007).

#### LasA Staphylolytic Assay

*Pseudomonas aeruginosa* uses *las* QS system which consists of *lasI* that interact with the LasR transcriptional activator to activate the *lasA* virulence gene which leads to the production of LasA protease. The ability of culture supernatant to lyse the boiled *Staphylococcus aureus* cells was determined by LasA protease activity (Kessler et al., 1993). A 30 ml overnight culture of *S. aureus* cells were centrifuged at 7,000 rpm for 3 min and the pellet was suspended in 0.02 M Tris-HC1 (pH 8.5). The suspension was boiled for 10 min and then diluted with the same buffer to an OD of 0.8 at 595 nm. To 900 μl of diluted *S. aureus* suspension, 100 μl of cell free culture supernatant of the strains cultured with or without EO was added. OD was measured at 595 nm after every 15 min interval for 60 min using a UV–visible spectrophotometer. Similarly, the LasA staphylolytic assay was carried out using cinnamaldehyde and linalool against *P. aeruginosa* and the clinical isolates.

#### Pyocyanin Assay

*Pseudomonas aeruginosa* uses *rhl* QS system that consists of *rhlI* which in conjunction with RhlR activates the expression of pyocyanin pigment production. Cell free supernatants of PA01 and the clinical isolates cultured with the presence and absence of EO at their respective sub-MIC concentrations (5 and 7% v/v) was extracted for pyocyanin quantification (Essar et al., 1990). The extracted pyocyanin was quantified spectrophotometrically at OD 520 nm. Cinnamaldehyde and linalool were also tested for their ability to inhibit pyocyanin produced by PA01 and the clinical isolates.

#### Swarming Motility

*Pseudomonas aeruginosa* uses QS to regulate several functions such as biofilm formation, motility, and pigment production. QS regulated swarming motility is characterized as a form of flagella dependent movement on a viscous environment such as semisolid agar surface (Krishnan et al., 2012). Initially, 10 ml of swarming agar [(1% w/v) glucose, (0.5% w/v) agar, (0.5% w/v) peptone and (0.2% w/v) yeast extract] was overlaid on to the sterile petri plate. The 5 ml of swarming media seeded with BIC of EO was poured as an additional layer and was allowed to solidify. The 2 μl of overnight grown *P. aeruginosa* cells (2 × 10^7^ CFU) was inoculated in the middle of the agar and incubated for 16 h at 37°C (Krishnan et al., 2012). Cinnamaldehyde and linalool were tested for its inhibition of swarming motility by the same method.

### Statistical Analysis

All experiments were carried out in triplicates. The statistical analogy among control and treated samples were determined using Graph Pad prism version 5. Multivariate analysis such as Tukey test was carried out to show the significance.

## Results

### Chemical Constituents of EO

GC-FID and GC-MS analysis of leaf EO of *C. tamala* enabled the identification of 40 volatile constituents amounting to 97.7% of the EO. Only two components namely, Linalool (42.5%) and (*E*)-cinnamaldehyde (31.2%) were the major compounds of the oil (**Table 1**).

**Table 1:** The chemical composition of essential oil extracted from *Cinnamomum tamala*.

S. No.	Compound	RI	Relative amount %
1	(2*E*)-Hexenal	852	0.1
2	α-Thujene	928	0.4
3	α-Pinene	937	3.1
4	Camphene	952	1.2
5	Benzaldehyde	962	1.3
6	β-Pinene	981	1.4
7	Myrcene	992	0.4
8	α-Phyllandrene	1004	0.8
9	δ-3-Carene	1013	0.4
10	ρ-Cymene	1027	3.6
11	Limonene	1032	0.9
12	1,8-Cineole	1035	0.6
13	Salicylaldehyde	1044	0.6
14	(*Z*)-Linalool oxide	1075	0.2
15	Terpinolene	1093	0.2
16	**Linalool**	**1100**	**42.5**
17	Benzenepropanal	1164	0.9
18	Borneol	1172	0.6
19	Terpinen-4-ol	1179	0.5
20	α-Terpineol	1189	0.2
21	(*Z*)-Cinnamaldehyde	1222	0.3
22	Nerol	1232	0.1
23	**(*E*)-Cinnamaldehyde**	**1272**	**31.2**
24	Bornyl acetate	1387	0.1
25	Thymol	1291	0.1
26	α-Ylangene	1378	0.2
27	α-Copaene	1380	0.1
28	Geranyl acetate	1384	0.1
29	(*Z*)-Cinnamyl acetate	1392	0.1
30	(*E*)-Caryophyllene	1422	1.0
31	Coumarin	1435	0.2
32	(*E*)-Cinnamyl acetate	1449	2.6
33	α-Humulene	1456	0.1
34	γ-Muurolene	1480	0.1
35	δ-Cadinene	1527	0.1
36	α-Cadinene	1537	0.1
37	α-Calacorene	1547	0.1
38	(*E*)-Nerolidol	1566	0.1
39	Spathulenol	1579	0.1
40	Caryophyllene oxide	1590	1.0
	**Total**		**97.7**


*Bolded values indicate the compounds present in high percentage.*

### Determination of Minimal Inhibitory Concentration of EO

The MIC of the EO against PAO1 and the he clinical isolates AU07 and AU09 was found to be 10 and 15% v/v from the various concentrations tested (1–20% v/v). The MIC of cinnamaldehyde against PAO1 was found to be 0.36 mg/ml and for clinical isolates it showed MIC of about 2 mg/ml. Likely, the MIC of linalool against PAO1 was found to be 0.36 mg/ml and for Clinical isolates it showed MIC of about 2 mg/ml. It was observed that chemical composition of volatile oils varied significantly among the different *Cinnamomum* sp. The major components of the EO obtained from the bark of Ceylon type *C. zeylanicum* were eugenol and (*E*)-cinnamaldehyde which represent 82.5% of the total composition (Dagli et al., 2015). Linalool was found to be the highest (42.5%) in underexplored *C. tamala.* As linalool is found to be a major component, we envisage that it might enhance the antimicrobial effect against *P. aeruginosa*. Previously, there was a report that EO in combination with linalool significantly enhances the antimicrobial effectiveness (Herman et al., 2016).

### Growth Curve Analysis

**FIGURE 1:**
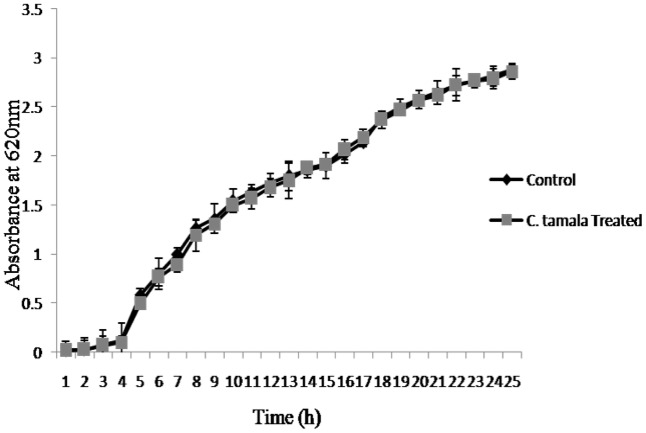
Growth curve of *P. aeruginosa* treated with EO of *C*. *tamala* after 24 h incubation.

Growth curve analysis was performed to ensure that the EO did not show any antibacterial activity at its sub-MIC (5%) concentration. The results no reduction in optical density of cells between control and treated even after 24 h of incubation (**Figure 1**).

### Inhibition of Young and Matured Biofilms

**FIGURE 2:**
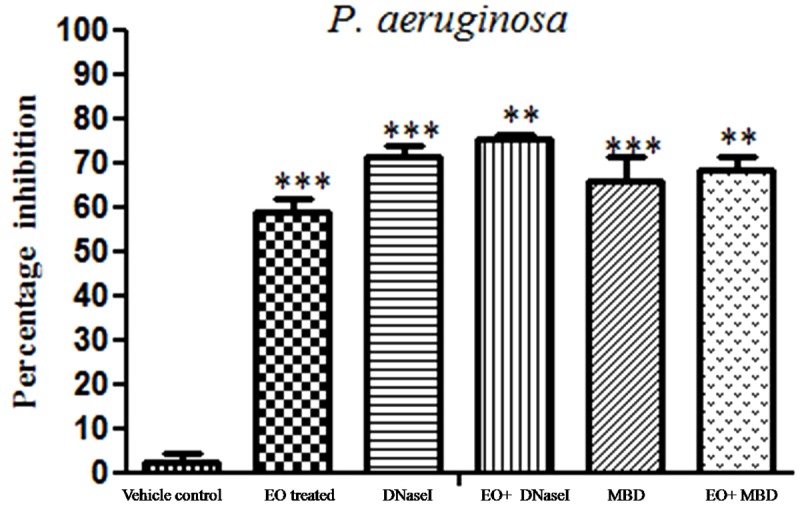
Percentage inhibition of *P. aeruginosa* young biofilm. Mean values of triplicate of independent experiments ± SD are shown. One-way ANOVA test demonstrates significant difference between the control and the test. Double asterisk indicates significant at *P* ≤ 0.01 and triple asterisk indicates significant at *P* ≤ 0.005.

**FIGURE 3:**
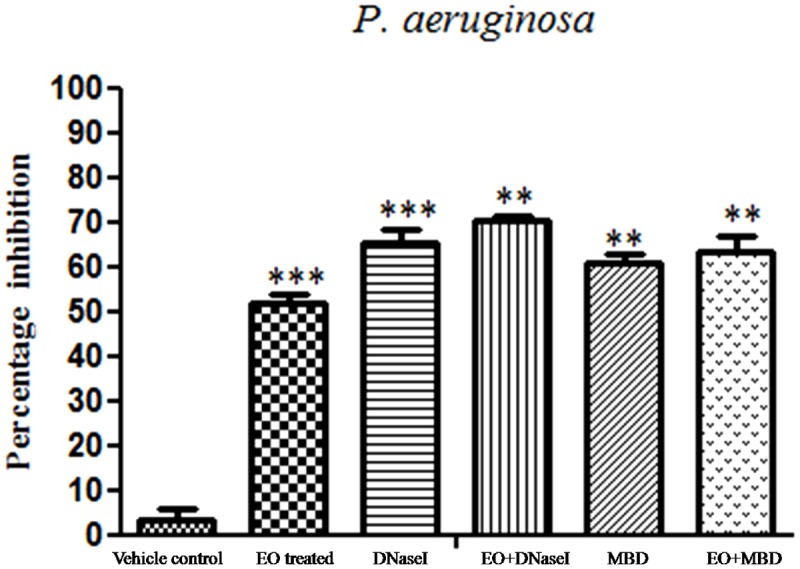
Percentage inhibition of *P. aeruginosa* matured biofilm. Mean values of triplicate independent experiments ± SD are shown. One-way ANOVA test demonstrates significant difference between the control and the test. Double asterisk indicates significant at *P* ≤ 0.01 and triple asterisk indicates significant at *P* ≤ 0.005.

The results showed that difference was observed in reduction of the biofilms treated with the combination of EO and DNases than EO and DNases treated individually. Biofilm inhibition of about 75% ± 1.09 was seen when EO and DNases were used in combination. On the other hand, EO showed only 58% ± 2.9 of inhibition when used individually (**Figure 2**). The results were in similar lines with matured biofilms. A maximum inhibition of about 70% ± 1.09 was seen in synergism of EO and DNases. EO alone on the other hand could inhibit only 50% ± 2.07 of the mature biofilm and 65% ± 2.8 inhibition was noticed for DNases alone (**Figure 3**).

### Microscopic Visualization of Antibiofilm Activity

#### Light Microscopy

**FIGURE 4:**
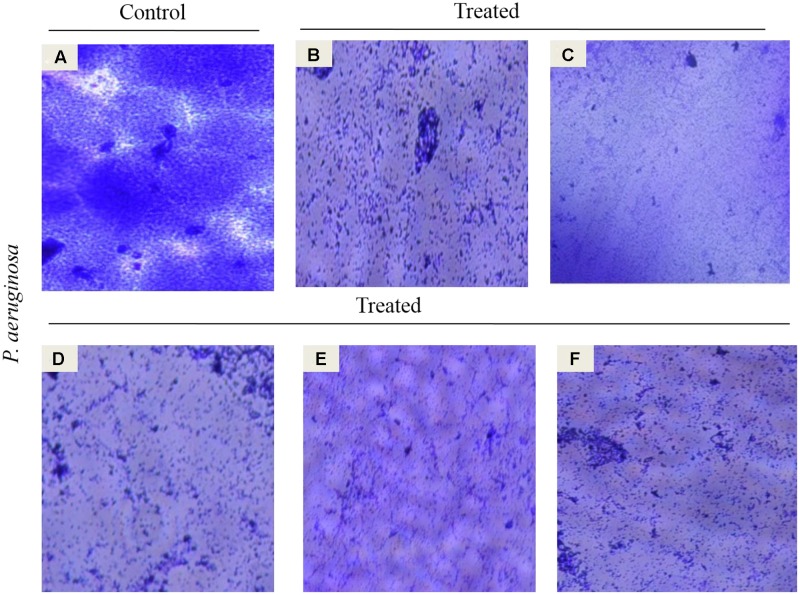
Light microscopic images at 40× demonstrate the inhibition of *P. aeruginosa* young biofilm. **(A)** Control, **(B)** EO treated, **(C)** DNaseI treated, **(D)** MBD treated, **(E)** EO + DNaseI, **(F)** EO + MBD.

To clearly demonstrate the synergized effect of EO and DNases on young biofilms, microscopic examinations were performed in the presence of sub-MIC (5% v/v) of EO. The microscopy analysis revealed maximum reduction of young biofilm upon treatment with EO and DNases. There was also reduction in EO treated biofilm but to a lesser extent on comparison with combination of EO and DNases (**Figure 4**).

#### Examination of Matured Biofilm Architecture by Confocal Laser Scanning Microscope

**FIGURE 5:**
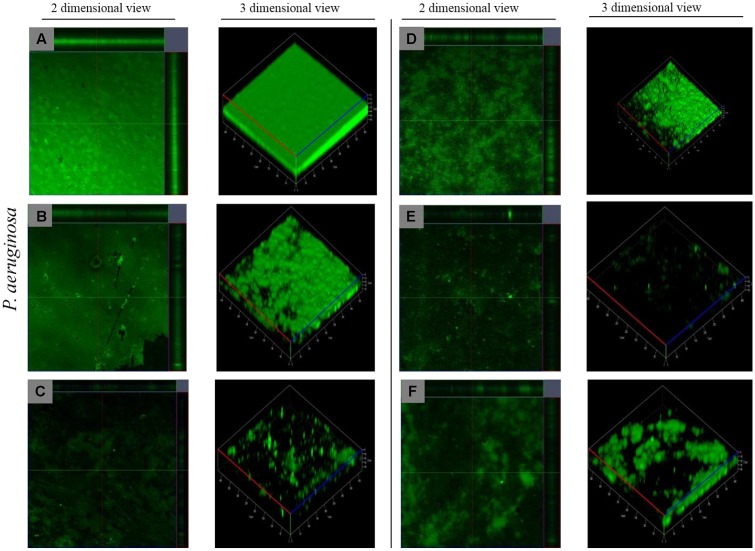
CLSM reveals the architecture of *P. aeruginosa* matured biofilm dispersal. **(A)** Control, **(B)** EO treated, **(C)** DNaseI treated **(D)** MBD treated, **(E)** EO + DNaseI, **(F)** EO + MBD.

Confocal Laser scanning microscopy was further done to confirm that synergized effect has maximum ability to disrupt matured biofilm than EO and DNases alone and to study the architecture of the biofilm before and after treatment. From the CLSM images (**Figure 5**), it is evident that there was a disintegration of biofilms in the treated samples on comparing with the control, which showed the presence of a dense biofilm. COMSTAT analysis was carried to determine the three dimensional features like biomass, average thickness and surface volume ratio of matured biofilms before and after treatment. The results show that there was a substantial reduction in different parameters of biofilm architecture such as biomass and average thickness, whereas there was an increase in surface to volume ratio which indicates the detachment of cells from biofilm matrix upon treatment (**Table 2**). From the **Table 2**, it is evident that the increase in surface to volume ratio (μm^2^/μm^3^) of around 0.043 and decrease in biomass (μm) (32.22) and average thickness (μm) (30.08) is due to the synergistic effect of both EO and DNase.

**Table 2:** COMSTAT analysis of treated and untreated *P. aeruginosa* biofilms.

Strain	Component	Biomass (μm)	Average thickness (μm)	Surface volume ratio (μm^2^/μm^3^)
	Control	85.09	82.77	0.021
	EO treated	62.89^∗∗∗^	60.12^∗∗∗^	0.026
*P. aeruginosa*	DNaseI	48.35^∗∗∗^	49.4^∗∗∗^	0.039
	EO + DNaseI	32.22^∗∗∗^	30.08^∗∗∗^	0.043
	MBD	53.01^∗∗∗^	49.13^∗∗∗^	0.028
	EO + MBD	42.67^∗∗∗^	40.12^∗∗∗^	0.031


*Mean values of triplicate individual experiments and SDs are shown. ^∗∗∗^Indicates the statistical significance (*P* < 0.05).*

#### Examination of Treated and Untreated *P. aeruginosa* Biofilm by SEM

**FIGURE 6:**
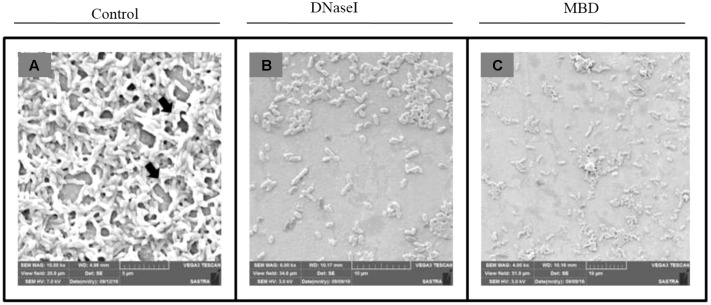
Scanning Electron Microscopic image of *P. aeruginosa* biofilm **(A)** Control, **(B)** DNaseI treated, **(C)** MBD treated. Black arrow in **(A)** control indicates the EPS forming matrix.

To analyze the antibiofilm potential of DNaseI and MBD on *P. aeruginosa* biofilm, SEM was performed. From the SEM results it is depicted that untreated *P. aeruginosa* (**Figure 6A**) has a thick layer of EPS, whereas there is a disruption in the EPS matrix of biofilm treated with DNaseI and MBD (**Figures 6B,C**).

#### Visualization of eDNA and effect of DNases on *P. aeruginosa* Biofilm

**FIGURE 7:**
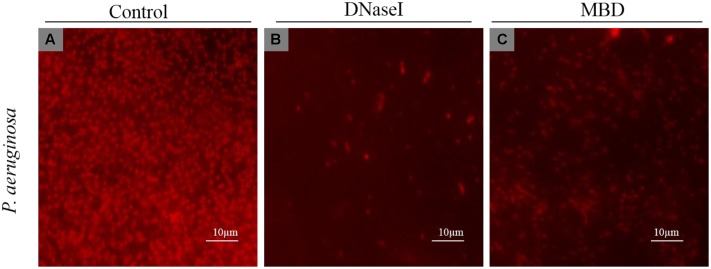
Fluorescent microscopy image of *P. aeruginosa* eDNA. Propidium iodide (PI) stained eDNA in red. **(A)** Control, **(B)** DNaseI treated, **(C)** MBD treated.

To detect the presence of eDNA, *P. aeruginosa* biofilms were stained with PI (which specifically stains eDNA) and was visualized under a fluorescent microscope. From the fluorescent micrograph results it is evident that untreated biofilm has high accumulation of eDNA (**Figure 7A**) whereas treated has lesser eDNA accumulation (**Figures 7B,C**).

### Virulence Assay

#### Inhibition of Alginate and EPS Production

**FIGURE 8:**
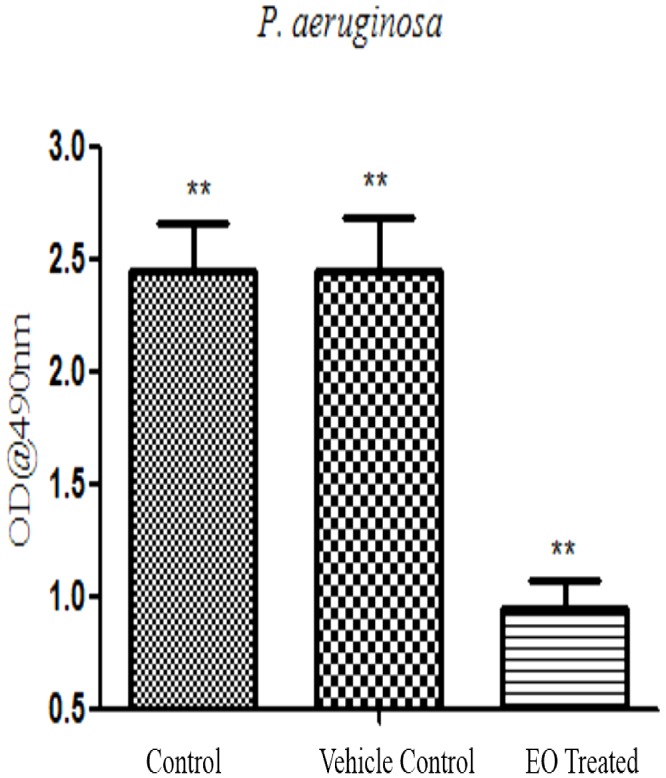
Inhibitory Effect of EO at sub MIC (5% v/v) on EPS production of *P. aeruginosa* biofilm. Mean values of triplicate independent experiments ± SD are shown. One-way ANOVA test demonstrates significant difference between the control and the test. Double asterisk indicates significant at *P* ≤ 0.01.

**FIGURE 9:**
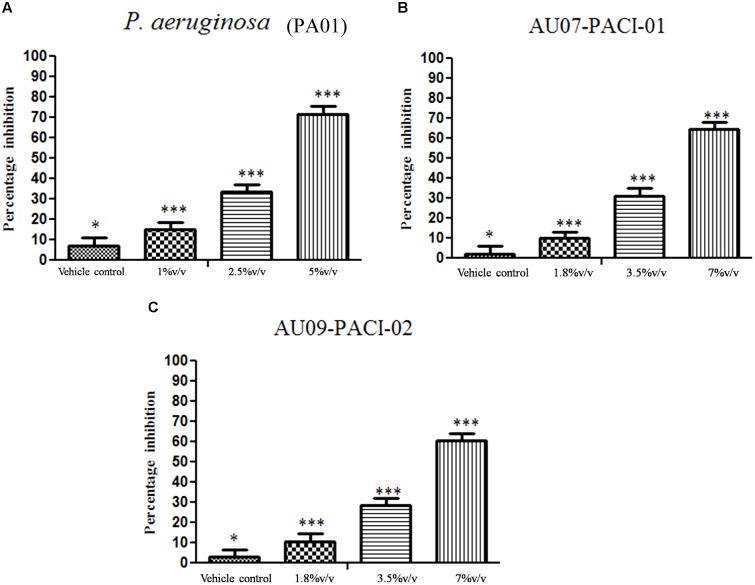
Inhibition of alginate production **(A)** *P. aeruginosa* biofilm in the presence of EO (1–5% v/v), **(B)** AU07-PACI-01 biofilm in the presence of EO (1.8–7% v/v), **(C)** AU09-PACI-02 biofilm in the presence of EO (1.8–7% v/v). Mean values of triplicate independent experiments ± SD are shown. One-way ANOVA test demonstrates significant difference between the control and the test. Single asterisk indicates significant at *P* ≤ 0.025 and triple asterisk indicates significant at *P* ≤ 0.005.

Essential oil inhibits the production of EPS, a maximum reduction in OD was observed with *P. aeruginosa* treated with sub-MIC (5% v/v) (**Figure 8**). Similarly, EO exhibited a concentration dependent inhibitory effect in the production of alginate by PAO1. At sub-MIC (5% v/v) the alginate production was reduced to the maximum of 71% ± 4.38 whereas 1% and 2.5% v/v had lesser inhibitory effect on alginate production (**Figure 9A**). The EO at its sub-MIC (7% v/v) inhibited the alginate production of clinical isolate AU07 by 65% ± 3.49 and 61% ± 3.58 for AU09 (**Figures 9B,C**). Likely, cinnamaldehyde inhibits the alginate production at its sub-MIC (0.18 mg/ml) of about 69% ± 2.75 for *P. aeruginosa*, 63.5% ± 4.63 for AU07 PACI-01 at its sub-MIC (1 mg/ml) and 59.5% ± 2.43 for AU09 PACI-02 at its sub-MIC (1 mg/ml) (Supplementary Figure S1). Linalool, another component of EO, inhibits alginate of about 67% ± 2.99 for *P. aeruginosa*, 63% ± 1.99 for AU07 PACI-01 and 60% ± 3.12 for AU09 PACI-02 (Supplementary Figure S2).

#### Effect of EO on LasA Staphylolytic Activity

**FIGURE 10:**
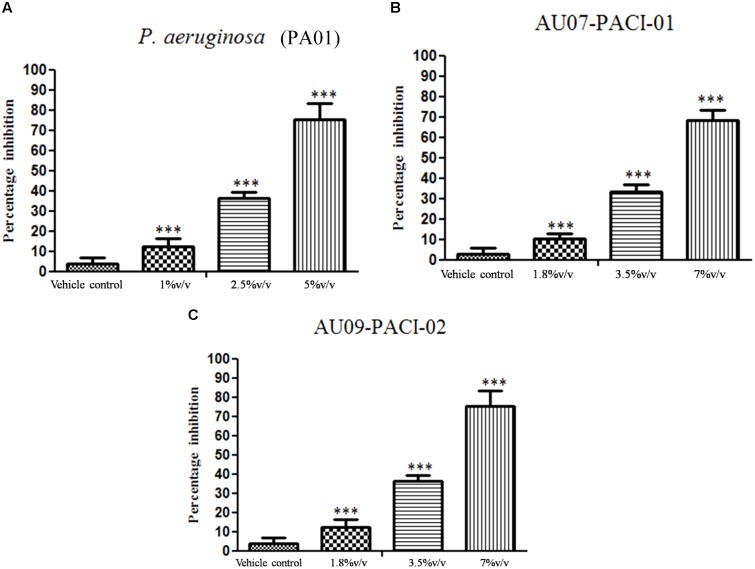
Inhibition of staphylolytic activity **(A)** *P. aeruginosa* biofilm by EO treated (1–5% v/v), **(B)** AU07-PACI-01 biofilm in the presence of EO (1.8–7% v/v), **(C)** AU09-PACI-02 biofilm in the presence of EO (1.8–7% v/v). Mean values of triplicate independent experiments ± SD are shown. One-way ANOVA test demonstrates significant difference between the control and the test. Single asterisk indicates significant at *P* ≤ 0.025 and triple asterisk indicates significant at *P* ≤ 0.005.

Supernatant of PAO1 treated with EO showed significant reduction in LasA staphylolytic activity in a concentration dependent manner. A maximum reduction of about 76% ± 8.06 was observed at sub-MIC (5% v/v) treated (**Figure 10A**). The EO at its sub-MIC (7% v/v) reduces the LasA staphylolytic activity for AU07 by 68% ± 4.95 and 75% ± 7.93 for AU09 (**Figures 10B,C**). The reduction of LasA staphylolytic activity was found to be 70% ± 5.26 for *P. aeruginosa* at its sub-MIC (0.18 mg/ml), 62.5% ± 4.22 for AU07 PACI-01 and 60% ± 3.99 for AU09 PACI-02 at its sub-MIC (1 mg/ml) (Supplementary Figure S3). For Linalool the reduction was found to be 67% ± 4.56 for *P. aeruginosa*, 63% ± 3.65 for *P. aeruginosa* and 60% ± 3.42 for AU09 PACI-02 (Supplementary Figure S4).

#### Effect of EO on Pyocyanin Production

**FIGURE 11:**
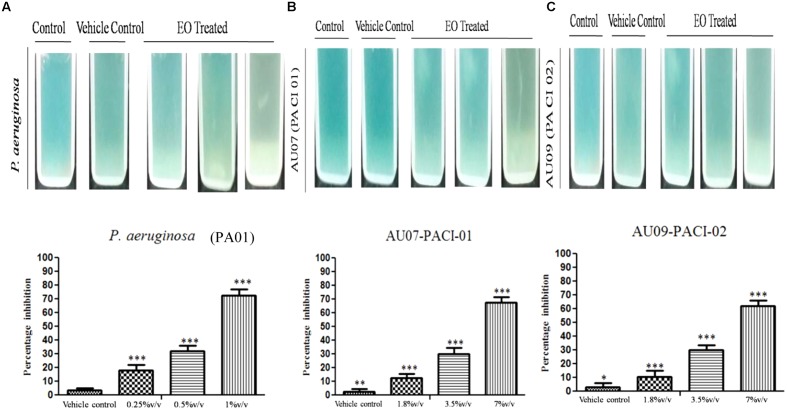
Inhibition of pyocyanin **(A)** *P. aeruginosa* biofilm by EO treated (1–5% v/v), **(B)** AU07-PACI-01 biofilm in the presence of EO (1.8–7% v/v), **(C)** AU09-PACI-02 biofilm in the presence of EO (1.8–7% v/v). Mean values of triplicate independent experiments ± SD are shown. One-way ANOVA test demonstrates significant difference between the control and the test. Single asterisk indicates significant at *P* ≤ 0.025, double asterisk indicates significant at *P* ≤ 0.01, and triple asterisk indicates significant at *P* ≤ 0.005.

The production of pyocyanin a blue green phenazine pigment in *P. aeruginosa* is regulated by QS system. *P. aeruginosa* biofilm treated with EO showed a maximum reduction of pyocyanin production of about 72% ± 4.65 at its sub-MIC (5%v/v) when compared to the control (**Figure 11A**). The EO at its sub-MIC (7% v/v) reduces the pyocyanin production by 68% ± 3.95 for AU07 and 62% ± 3.90 for AU09 (**Figures 11B,C**). Pyocyanin was reduced significantly of about 78% ± 4.70 at its sub-MIC (0.18 mg/ml) for *P. aeruginosa* treated with cinnamaldehyde and 67% ± 0.87 for AU07 PACI-01 at its sub-MIC (1 mg/ml) and 63% ± 2.01 for AU09 PACI-02 at its sub-MIC (1 mg/ml) (Supplementary Figure S5). Likely, linalool significantly reduces pyocyanin pigment of about 70% ± 2.94 for *P. aeruginosa*, 67% ± 1.30 for AU07 PACI-01 and 65% ± 2.63 for AU09 PACI-02 (Supplementary Figure S6).

#### Effect of EO on *P. aeruginosa* Swarming Motility

**FIGURE 12:**
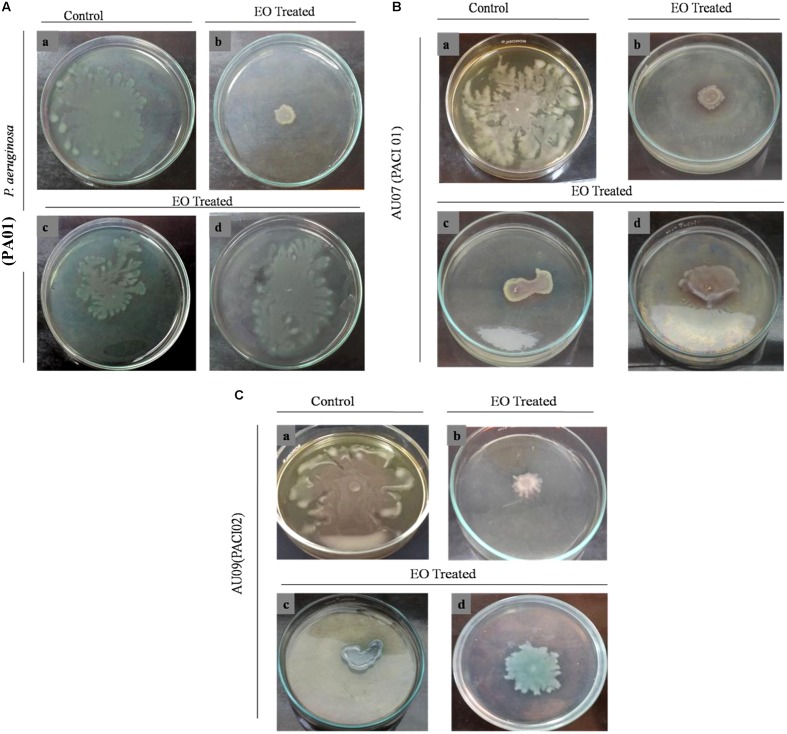
Effect of EO on swarming motility of **(A)** *P. aeruginosa* in a concentration dependent manner. **(a)** Control, **(b)** EO Treated (1.25% v/v), **(c)** (2.5% v/v), **(d)** (5% v/v). **(B)** AU07-PACI-01 **(a)** Control, **(b)** EO Treated (1.8% v/v), **(c)** (3.5% v/v), **(d)** (7% v/v). **(C)** AU09-PACI-02 **(a)** Control, **(b)** EO Treated (1.8% v/v), **(c)** (3.5%v/v), **(d)** (7% v/v).

To explore the anti-QS potential of the EO of *C. tamala*, swarming assay was done against *P. aeruginosa* in a concentration dependent manner. Maximum reduction of swarming motility was observed in the swarming plates treated with EO at its sub-MIC (5% v/v) concentration (**Figure 12**). From the results, it is envisaged that EO at its BIC (5% v/v) has the ability to interfere in the *Las* and *Rhl* mediated QS system. The individual component of EO such as cinnamaldehyde (Supplementary Figure S7) and linalool (Supplementary Figure S8) inhibit swarming motility of *P. aeruginosa* and two clinical isolates such as AU07 PACI-01 and AU09 PACI-02 at its sub-MIC. Earlier studies which report about the anti-QS activity of cinnamon oil cite that cinnamaldehyde was the major component of the oil and it was responsible for the anti-QS activity (Brackman et al., 2008; Yap et al., 2015). However, a recent study which report about the anti-QS activity of Ceylon-type whole cinnamon oil had eugenol as its major component (Kalia et al., 2015). In similar lines, the EO from *C. tamala* had linalool (42%) as its major component followed by cinnamaldehyde (31%). So, we fully concur with the view that the exhibited QSI activity might be due to the synergistic action of the major components present in the EO (Kalia et al., 2015). The potency of an EO form an underexplored *Cinnamomum* sp. in attenuating QS dependent swarming motility provides a new insight into the anti-virulent potential of the EO and opens up a new avenue for bioprospecting EOs from several underexplored wild *Cinnamomum* sp. present in the Himalaya range.

## Discussion

As biofilms are resilient and polymicrobial (Stewart, 2015), a combination of approaches is needed for the development of antibiofilm drugs. Owing to their different modes of action, combination strategies open up new avenues for novel antibiofilm pharmaceuticals. In the present study, we show that EOs when combined with DNase show enhanced disruption of biofilms. A recent study reports that the sole use of EO (*C. zeylanicum)* alone showed very low efficacy in reducing *P. aeruginosa* biofilms (Kalia et al., 2015). The present study demonstrated that the synergistic effect of EO and DNase inhibit biofilms to a greater extent. Several synergistic studies have been carried out by using DNase and antibiotics in tandem which show that the removal of eDNA weakens the biofilm matrix (Conover et al., 2011) and thereof increases the susceptibility of both Gram-positive and -negative pathogens to antibiotics (Tetz et al., 2009; Nguyen and Burrows, 2014). Similarly, the synergistic effects of various EOs and antibiotics have also shown enhanced biofilm removal (Duarte et al., 2012; Coelho and Pereira, 2013; Lang et al., 2016). On this basis, the present study shows that synergism of the EO of *C. tamala* with DNases can also be an effective means to treat *P. aeruginosa* biofilm infections. The MBD was also more efficient in disrupting the mature biofilms than the EO alone. This shows that EOs have limited penetration into the biofilms and when the biofilm scaffold which includes eDNA is loosened due to the degradation of eDNA by the action of DNAses it enhances the action of EOs. Chronic infection caused by biofilm forming strains of *P. aeruginosa* during cystic fibrosis (CF) mediates a neutrophil dominated host immune response. During this process a very high concentration of e-DNA gets released as neutrophils disintegrate which leads to increased sputum accumulation (Bayes et al., 2016). Recombinant DNase is reported to reduce this copious amount of viscous sputum, thereby clearing the lower airway secretions (Pressler, 2008). On this basis, the present study shows that synergism of EO of *C. tamala* with DNases can be an effective way to disrupt *P. aeruginosa* biofilm associated infections. Alginate is one of the major compounds present in the EPS layer of *P. aeruginosa* biofilms which helps to maintain the structural integrity of biofilms. In the present study, we observed that *C. tamala* significantly inhibited alginate production.

Of late, EOs of different plants has been documented to have QS inhibitory activity (Khan et al., 2009; Kalia et al., 2015; Pekmezovic et al., 2016). In the present study, we observed that the EO from *C. tamala* effectively suppressed several QS mediated virulence factors related to *P. aeruginosa*. It was interesting to note that the EO was also able inhibit the virulence factors of even the clinical isolates of *P. aeruginosa* which showcases the anti-infective ability of EOs from native plants and stresses the need for bioprospecting native plant species for anti-infectives. *P. aeruginosa* possesses two distinct, but interacting, well-characterized QS systems namely *las* (LasI, LasR) and *rhl* which coordinates several extracellular virulence factors (Krishnan et al., 2012). From the results, it is suggested that inhibition of pyocyanin production might be due to the presence of LasR or *rhl* inhibitor in EO (**Figure 11**). Swarming motility is a key virulent trait in *P. aeruginosa* which is mediated by *Las* and *Rhl* QS system (Pesci et al., 1997; Kumar et al., 2009) which plays a crucial role in dissemination of the pathogen. From the results it was evident that the EO was able to disrupt the dissemination or group motility of the pathogen a crucial factor in biofilm formation. We further show that cinnamaldehyde and linalool, the major compounds in the EO of *C. tamala* suppressed the QS-based virulence factors in *P. aeruginosa* PAO1 as well as the clinical isolates. Cinnamaldehyde is already reported as a QS inhibitor in *V. harveyi* probably by inhibiting/degrading the QS molecule 3-hydroxy-butanoyl homoserine lactone. On the other hand 3-oxo-C_12_HSL and C_4_HSL are the major QS molecules in *P. aeruginosa* (Kalia et al., 2015). So it is envisaged that the QS inhibitory mechanism by cinnamaldehyde and linalool is possibly mediated by acting upon the major QS molecules in *P. aeruginosa*.

Though several EOs have been explored for their antibiofilm property, to best of our knowledge, the present study forms a first report about the QS and biofilm inhibitory potential of an underexplored EO of *C. tamala* and the synergistic combination of EO of *C. tamala* and DNases has increased efficacy in disrupting biofilms. The synergistic use of EO with a DNase of marine bacterial origin might result in a cost effective therapy and its use as a potential antibiofilm agent for the treatment of biofilm device associated infections warrants further investigations.

## Author Contributions

All authors listed have made a substantial, direct and intellectual contribution to the work, and approved it for publication. PN designed overall research and wrote the paper.

## Conflict of Interest Statement

The authors declare that the research was conducted in the absence of any commercial or financial relationships that could be construed as a potential conflict of interest.
