# Supplementary material for: Antivirulent Properties of Underexplored Cinnamomum tamala Essential Oil and Its Synergistic Effects with DNase against Pseudomonas aeruginosa Biofilms – An In Vitro Study
Source: Front Microbiol. 2017 Jun 26;8:1144. doi: 10.3389/fmicb.2017.01144 (PMC5483474; doi:10.3389/fmicb.2017.01144)

**Supplementary Figure Legends**

**FIGURE S1**. Alginate production inhibition by Cinnamaldehyde (A) *P. aeruginosa*, (B) AU07- PACI- 01, (C) AU09- PACI- 02. Mean values of triplicate independent experiments ± SD are shown. One way ANNOVA test demonstrates significant difference between the control and the test. Triple asterisk indicates significant at P≤0.005.

**FIGURE S2**. Alginate production inhibition by Linalool (A) *P. aeruginosa*, (B) AU07- PACI- 01, (C) AU09- PACI- 02. Mean values of triplicate independent experiments ± SD are shown. One way ANNOVA test demonstrates significant difference between the control and the test. Triple asterisk indicates significant at P≤0.005.

**FIGURE S3**. Inhibition of LasA staphylolytic activity by Cinnamaldehyde (A) *P. aeruginosa*, (B) AU07- PACI- 01, (C) AU09- PACI- 02. Mean values of triplicate independent experiments ± SD are shown. One way ANNOVA test demonstrates significant difference between the control and the test. Triple asterisk indicates significant at P≤0.005.

**FIGURE S4**. Inhibition of LasA staphylolytic activity by Linalool (A) *P. aeruginosa*, (B) AU07- PACI- 01, (C) AU09- PACI- 02. Mean values of triplicate independent experiments ± SD are shown. One way ANNOVA test demonstrates significant difference between the control and the test. Triple asterisk indicates significant at P≤0.005.

**FIGURE S5**. Pyocyanin reduction by Cinnamaldehyde (A) *P. aeruginosa*, (B) AU07- PACI- 01, (C) AU09- PACI- 02. Mean values of triplicate independent experiments ± SD are shown. One way ANNOVA test demonstrates significant difference between the control and the test. Triple asterisk indicates significant at P≤0.005.

**FIGURE S6**. Pyocyanin reduction by Linalool (A) *P. aeruginosa*, (B) AU07- PACI- 01, (C) AU09- PACI- 02. Mean values of triplicate independent experiments ± SD are shown. One way ANNOVA test demonstrates significant difference between the control and the test. Triple asterisk indicates significant at P≤0.005.

**FIGURE S7**. Inhibition of swarming motility by Cinnamaldehyde (A) *P. aeruginosa*- a) Control, b) 0.18mg/ml treated, c) 0.09mg/ml treated, d) 0.045mg/ml treated. (B) AU07- PACI- 01- a) Control, b) 1mg/ml treated, c) 0.5mg/ml treated, d) 0.25mg/ml treated. (C) AU09- PACI- 02- a) Control, b) 1mg/ml treated, c) 0.5mg/ml treated, d) 0.25mg/ml treated.

**FIGURE S8**. Inhibition of swarming motility by Linalool (A) *P. aeruginosa*- a) Control, b) 0.18mg/ml treated, c) 0.09mg/ml treated, d) 0.045mg/ml treated. (B) AU07- PACI- 01- a) Control, b) 1mg/ml treated, c) 0.5mg/ml treated, d) 0.25mg/ml treated. (C) AU09- PACI- 02- a) Control, b) 1mg/ml treated, c) 0.5mg/ml treated, d) 0.25mg/ml treated.

Figure S1. TIF


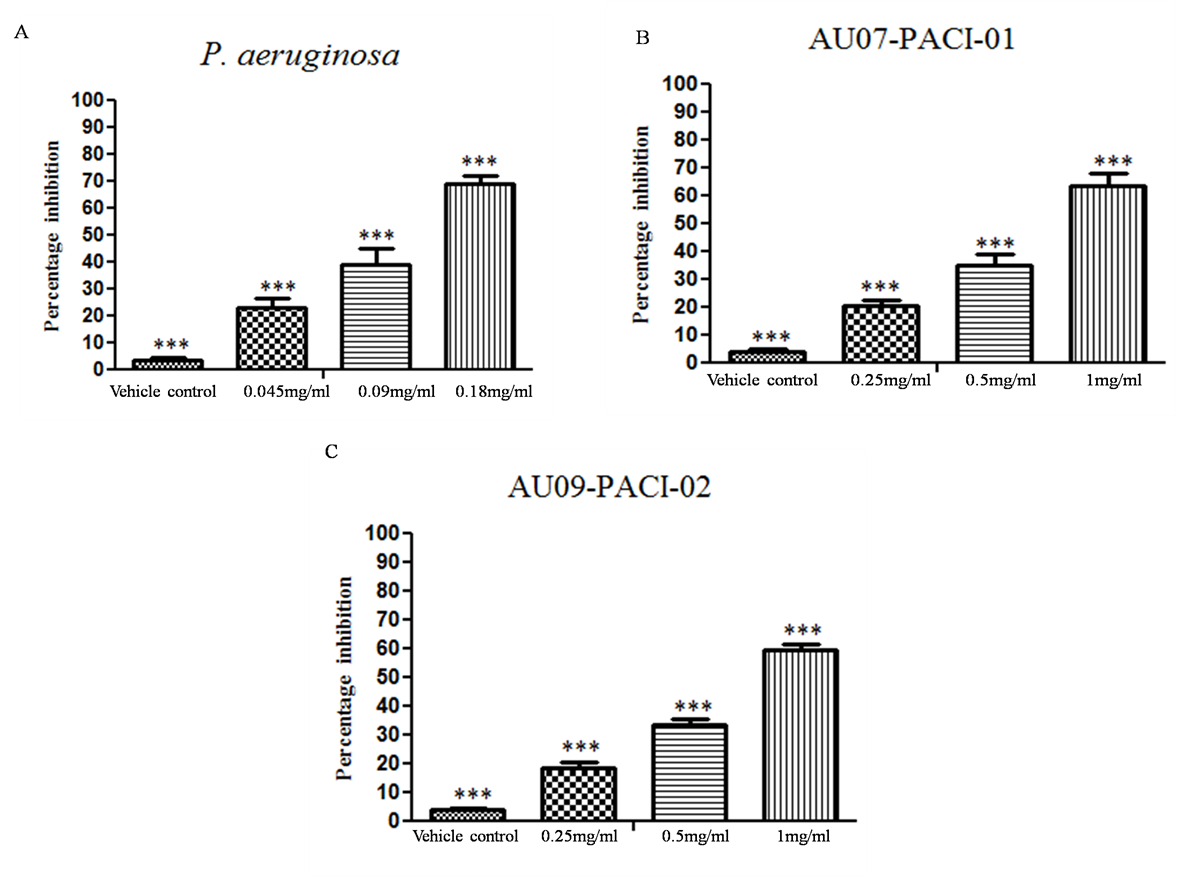


Figure S2. TIF


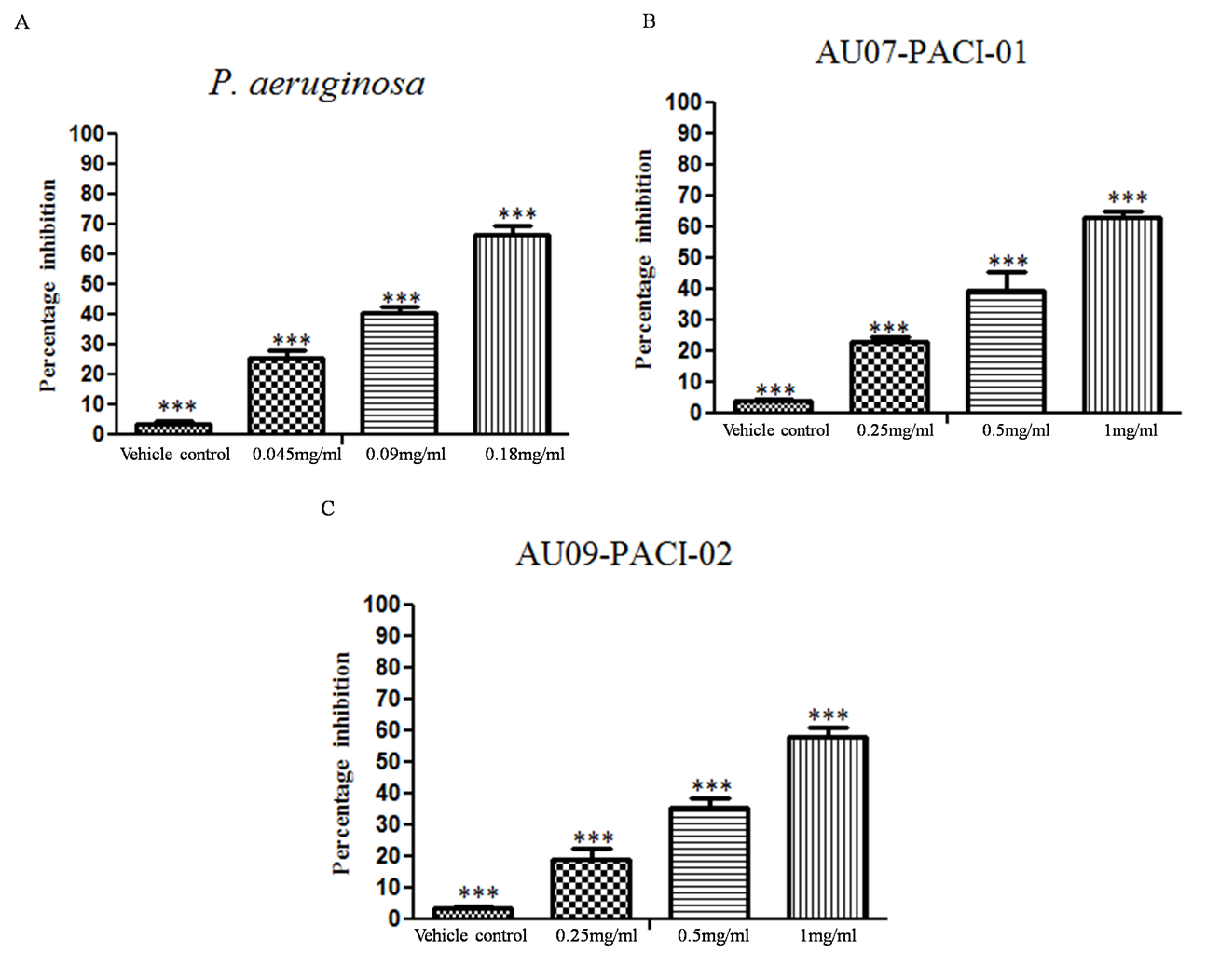


Figure S3. TIF


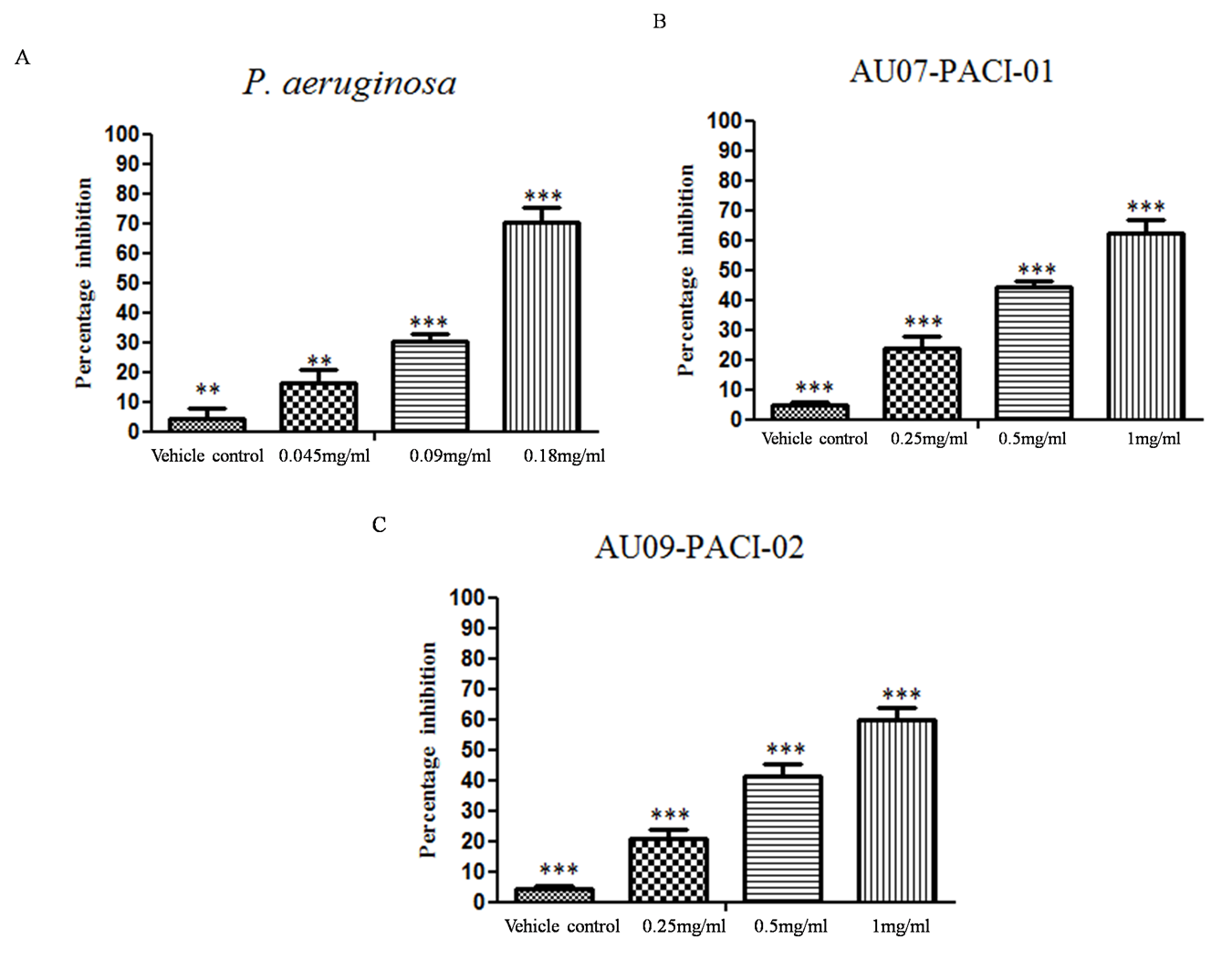


Figure S4. TIF


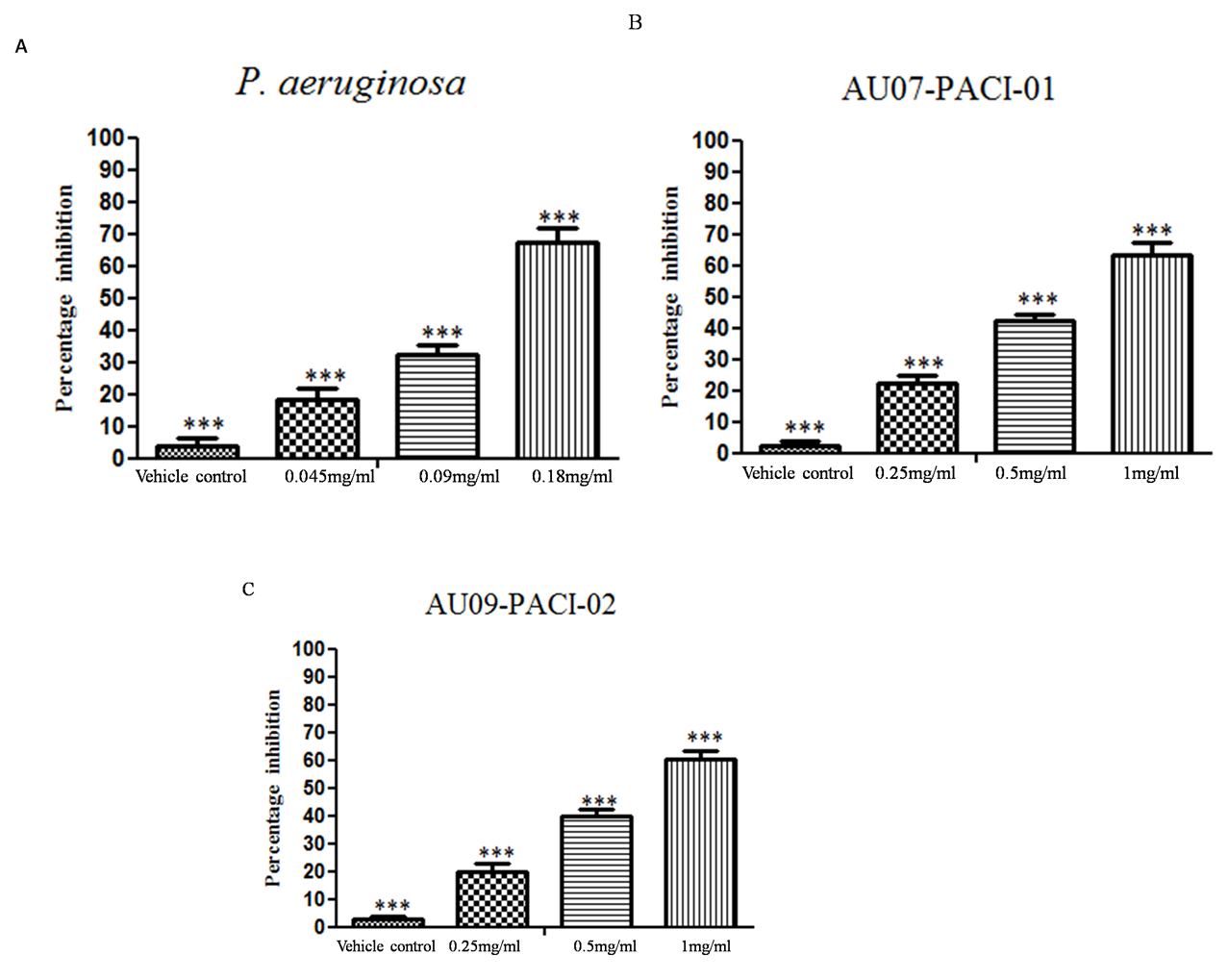


Figure S5. TIF


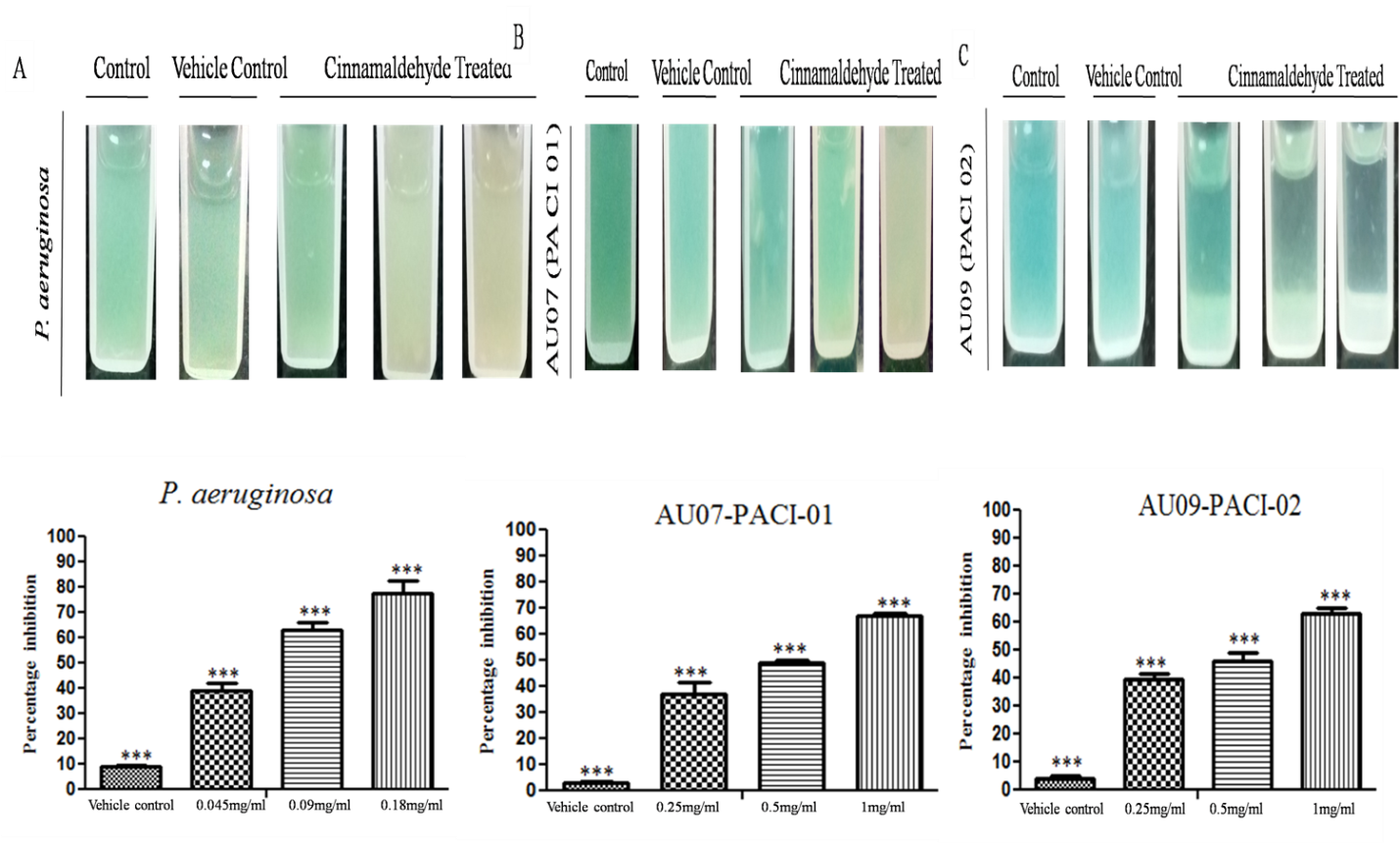


Figure S6. TIF


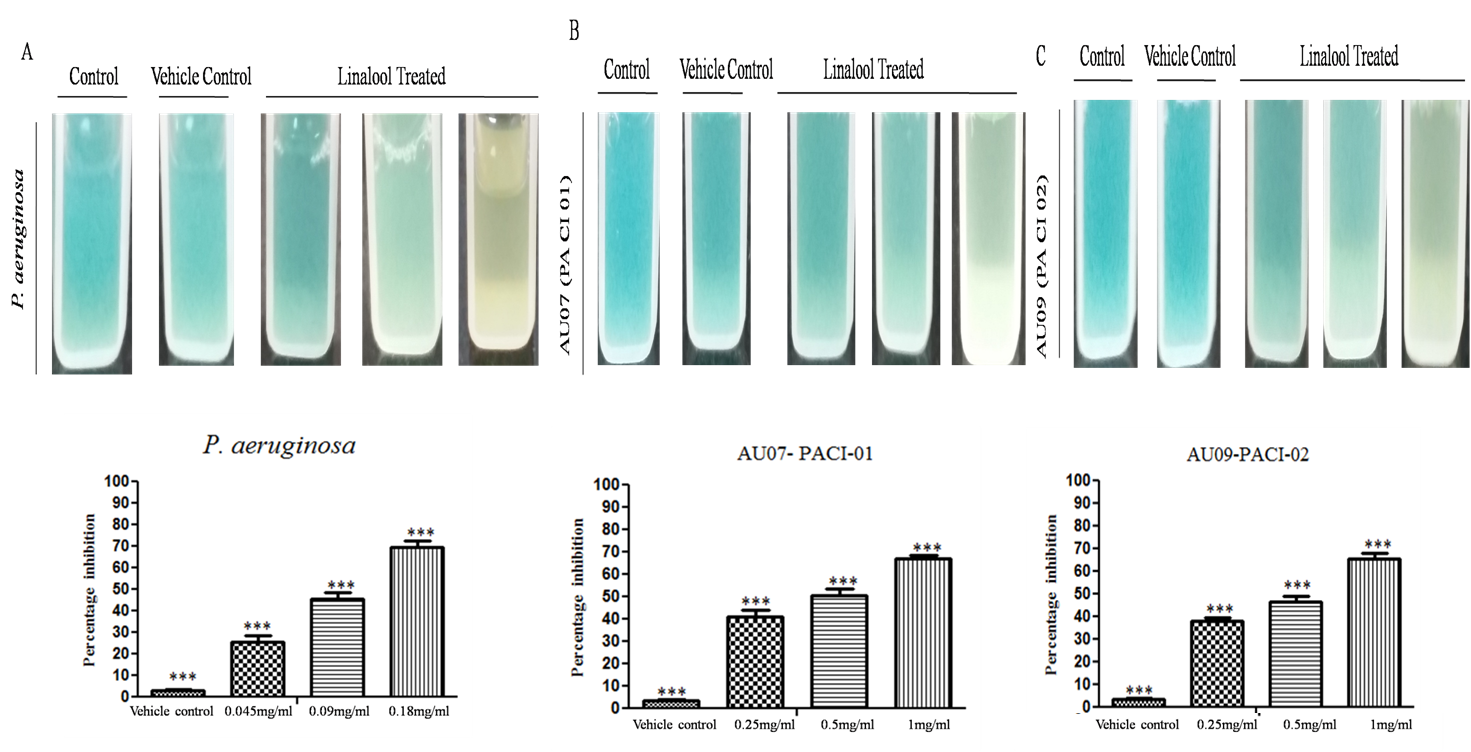


Figure S7. TIF


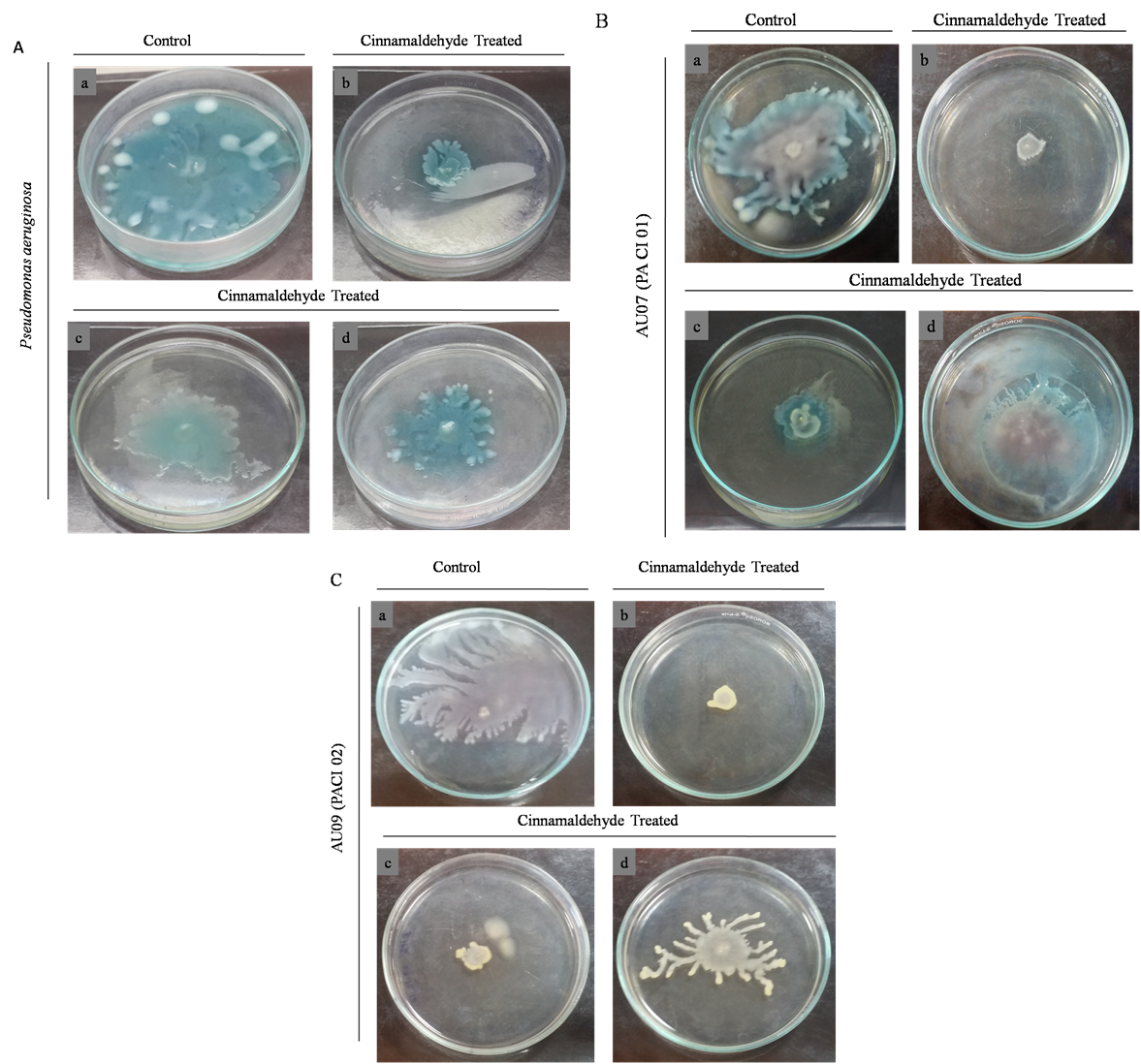


Figure S8. TIF


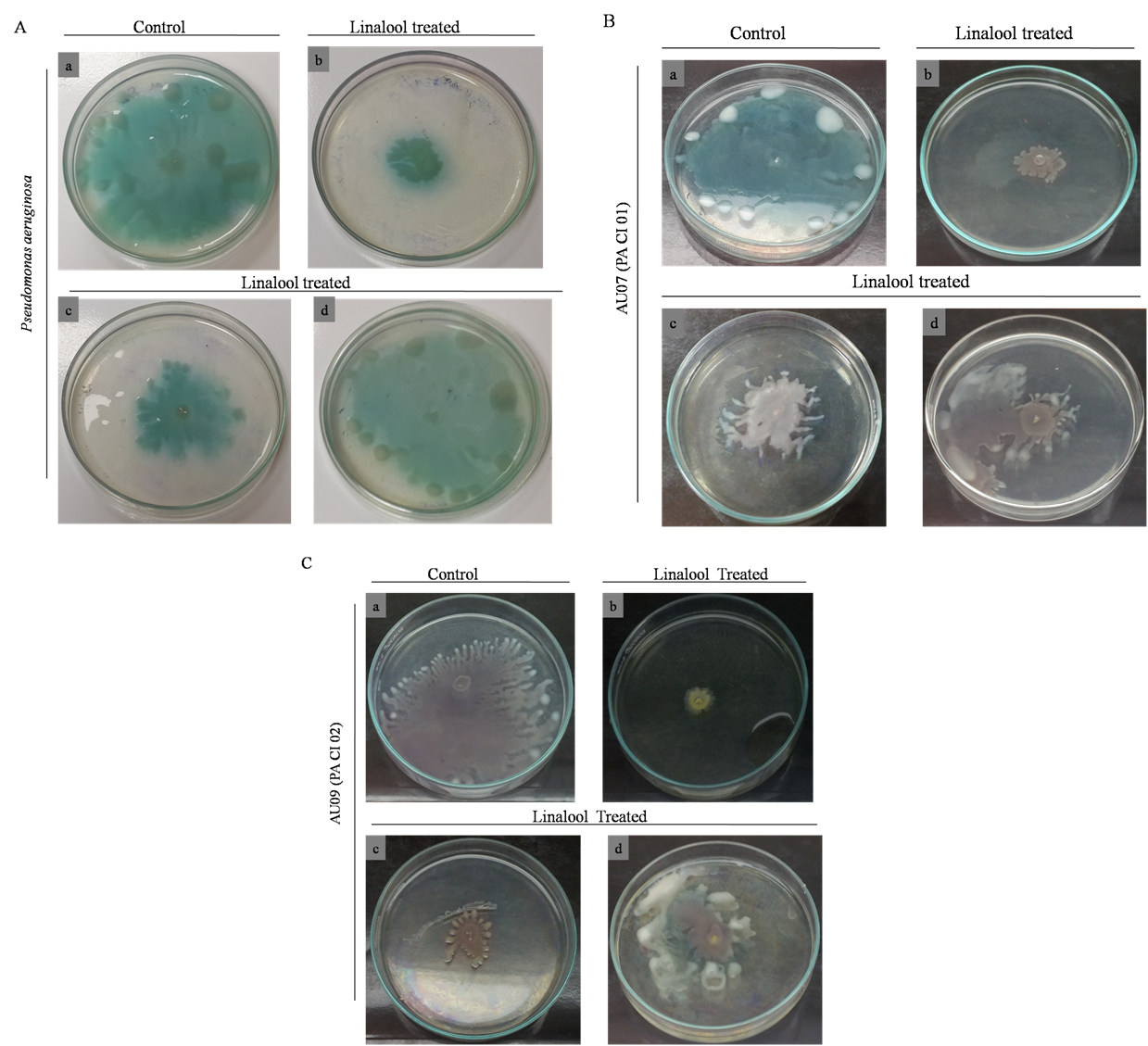

Supplement: Supplementary file 1 [file Data_Sheet_1.docx]
